# İnter-rater and intra-rater reliability of the extended TUG test in elderly participants

**DOI:** 10.1186/s12877-020-1460-0

**Published:** 2020-02-13

**Authors:** Juan José Bedoya-Belmonte, María del Mar Rodríguez-González, Manuel González-Sánchez, Jose Miguel Barreda Pitarch, Alejandro Galán-Mercant, Antonio I. Cuesta-Vargas

**Affiliations:** 1 Servicio Andaluz de Salud, Distrito Malaga, CS tiro pichón, Málaga, Spain; 2 Departamento de Fisioterapia, Grupo de Clinimetría (F-14), Facultad de Ciencias de la Salud, Instituto de Biomedicina de Málaga (IBIMA), Andalucía Tech, Universidad de Málaga, Arquitecto Francisco Peñalosa s/n. (ampliación Campus Teatinos), 29071 Málaga, Spain; 3 Departamento de Ciencias de la Salud, Facultad de Ciencias de la Salud, Universidad de Jaén, Jaén, Spain; 4 Departamento de Fisioterapia; Facultad de Enfermería y Fisioterapia, Universidad de Cádiz, Cádiz, Spain; 5 School of Clinical Sciences of the Faculty of Health, Queensland University of Technology, Brisbane, Australia

## Abstract

**Background:**

To analyse the reliability, variance and execution time of the Extended Timed Up and Go (Extended TUG) test in three age groups of elderly participants (G1: 55–64 years; G2: 65–74 years; G3: 75–85 years).

**Methods:**

An analytical cross-sectional study of 114 recruited participants (63 women) of average age 70.17 (± 7.3) years was undertaken. Each participant performed the Extended TUG three consecutive times, with a rest break between tests of 120 s. Both the intragroup and intergroup reliability of the measurements in the Extended TUG were analysed.

**Results:**

The reliability of the Extended TUG test is excellent for the first and second decades but drops down to good for the third decade. Specifically, intragroup reliability ranged from 0.784 for G3 to 0.977 for G1 (G2 = 0.858). Intergroup reliability, compared with intragroup reliability, was slightly lower, ranging between 0.779 for G3 and 0.972 for G1 (G2 = 0.853).

**Conclusion:**

The reliability of the Extended TUG test progressively decreases with increasing age, being excellent for the younger age groups and good for the oldest age group.

## Introduction

The world’s population is experiencing a gradual and incessant increase in the number of elderly people [1]. The frailty associated with aging has been studied for decades [2]. In the last two decades, the concept of frailty has undergone a considerable change, associated with the development of epidemiological studies on population aging [3–5]. These studies have allowed us to explain the frailty phenotype in a more adequate and empirical way, as a situation of biological instability related to the aging of human beings [5–8].

Currently, the early identification of frailty is centred on the loss of functional capacities, comorbidities, the appearance of disability and dependencies, etc. [9, 10] Early detection of the particular situations that lead to the dependence of elderly people will enable the establishment of corrective measures to prolong an individual’s autonomy [11].

Among the depletions associated with aging and frailty is a decrease in the speed of walking [12]. The assessment of gait speed has been shown to be a reliable marker, both for assessing survival and for predicting adverse events in the elderly (falls, hospitalization, need for caregivers, etc.) [12]. A slow gait velocity in healthy seniors acts as a predictor of adverse events, the early detection of which would favour priority interventions that could improve their physical condition and quality of life [13, 14]. There is previous scientific literature that reliably identifies an exact calculation of this gait speed, which has recently become a validated test in our environment as a diagnostic tool for frailty [15–19].

One of the functional tests most frequently used to analyse the characteristics of the functional gait is the Extended Timed Up and Go (Extended TUG). As the path taken in the Extended TUG is longer (10 m), it allows better analysis of the kinematic variables extracted during ambulation compared to the classic TUG [1]. The Extended TUG is highly correlated with the pure measures of the speed of walking and seems to be a very useful measure to predict health outcomes because it requires additional skills such as leg strength, balance and coordination [18–21]. Although the Extended TUG is used routinely in the assessment of mobility and function of the elderly, no study has been found that analyses the reliability of this test by dividing the participants into three age groups (G1: 55–64 years; G2: 65–74 years; G3: 75–85 years).

## Material and methods

### Aim

The main objective of the present study is to analyse the reliability (intragroup and intergroup) of the Extended TUG test in three groups of healthy adult participants (G1 decade: 55–64 years; G2 decade: 65–74 years; G3 decade: 75–85 years). Another objective of this study is to analyse the variance between the three study groups described above and to analyse how the execution of the Extended TUG test evolves over the years.

### Design and participants

This was an analytical cross-sectional study. A total of 114 participants (63 women, 51 men) of average age 70.17 years (SD = 7.3 years) were recruited from a public health centre and divided into three age groups (G1 decade: 55–64 years; G2 decade: 65–74 years; G3 decade: 75–85 years).

Exclusion criteria were: a score on the scale of assessment of the basic activities of Barthel’s daily life of less than 90; or the presence of diagnoses that indicate neuromuscular, metabolic, hormonal and/or cardiovascular alterations that contraindicate performing physical exercise [22–24].

The Research Ethics Committee of the University of Málaga approved the current study. The personal data of the participants were protected according to the Organic Law of Protection of Personal Data 19/55. The study was carried out according to the principles of the Declaration of Helsinki to guarantee protection of the rights, safety and well-being of the participants. All participants were verbally informed about the study and submitted signed informed consent before beginning their participation in this study.

### Procedures

#### The extended TUG test

The Extended TUG is a test that allows one to analyse the speed of the functional gait of a participant [24]. This test should be performed as quickly as possible but without running. The time that each participant needs to get up from a chair without armrests, walk for 10 m, make a 180° turn around a cone, return to the starting chair and sit again is the basis of the test [24].

Once the test was explained, each participant was able to perform it as many times as they deemed appropriate until complete understanding and correct execution was guaranteed. After this period of familiarization and a subsequent rest of 300 s, each participant performed two series of three repetitions each. The rest between each repetition was 120 s whereas the rest between each series was 10 min. Both series were supervised by a different clinical professional with more than 10 years of experience in the application of this functional test. The repetition that was done faster (less time recorded) was used for statistical analysis of the sample. In addition, by using the results from the first and second series, intragroup and intergroup analysis of the reliability of the measurement was carried out.

There were two outcome variables of the present study: the time needed to complete the Extended TUG test by the participants; and the reliability of the results calculated for each participant.

### Statistical analysis

Descriptive analysis of the sample was carried out both globally and adjusted for the decades (G1 decade: 55–64 years; G2 decade: 65–74 years; G3 decade: 75–84 years). The Kolmogorov-Smirnov test was performed to determine the distribution of all study variables. Analysis of the intragroup and intergroup reliability of the measurements in the Extended TUG test for each of the decades was performed using the test-retest method, with an interclass correlation (ICC) of 2:1. Reliability was classified as follows: ICC ≤ 0.40 (poor); 0.60 > ICC > 0.40 (moderate); 0.80 > ICC ≥ 0.60 (good); ICC ≥ 0.80 (excellent) [25]. The different groups were compared for both the descriptive and outcome variables, using Student’s *t*-test for the parametric variables and the Wilcoxon test for non-parametric variables. In addition, the reliability values for the different decades (intergroup analysis) were compared. The level of significance was established at *p* ≤ 0.05. The SPSS program (V.21) was used to carry out the statistical analysis.

## Results

The Kolmogorov-Smirnov test revealed that the distribution of the sample was non-parametric in all cases, except for the reliability of the measurements obtained.

Table 1 shows the anthropometric data of the sample, in measures of central tendency and dispersion, for all the groups together and also for each of the separate decades.

**Table 1:** Anthropometric data and values of the extended functional gait (MFe) (*n* = 114). Mean (± SD)

Variables	Decade 1 (55–64 years)	Decade 2 (65–74 years)	Decade 3 (75–85 years)
Edad Media (years)	**60.07** (±2.22)	**70.04** (±2.88)	**78.38** (±3.24)
Weight (Kg)	**72.42** (±14.91)	**72.56** (±14.75)	**72.72** (±11.49)
Height (meters)	**1.59** (±0.79)	**1.55** (±0.08)	**1.56** (±0.08)
BMI (Kg/m^2^)	**29.83** (±5.85)	**30.07** (±4.09)	**29.88** (±3.78)
Time (mean) (Seconds)	**14.49** (±5.11)	**17.29** (±13.87)	**20.53** (±15.09)
**N**	**37**	**42**	**34**

Among the anthropometric variables, when comparing all the groups significant differences were observed for age (between all the decades) and for height between decades G1 and G2 (*p* < 0.05). However, no significant differences were observed between the groups for the other anthropometric variables. Comparison of the execution time of the Extended TUG test between the groups revealed that there were significant differences (*p* ≤ 0.05) between all the groups (G1 vs. G2; G2 vs. G3; G1 vs. G3) (Table 1).

Table 2 shows the mean values of intragroup and intergroup reliability, as well as the values of the significance of the results obtained when comparing the different decades. Table 2 shows how the reliability of the Extended TUG test is excellent for the first and second decades but drops to good for the third decade [25]. When comparing the reliability between the three decades, significant differences were observed in all comparisons. However, when comparing intragroup and intergroup reliability within each decade, no significant differences were observed (Table 2).

**Table 2:** Results of the intragroup and intergroup reliability analysis and the differences between the different decades. ICC mean value and standard deviation

Variables	Decade 1 (55–64 years)	Decade 2 (65–74 years)	Decade 3 (75–85 years)	Significance D1-D2	Significance D1-D3	Significance D2-D3
Intragroup	**0.977** (±0.012)	**0.858** (±0.017)	**0.784** (±0.020)	**0.013**	**≤0.001**	**≤0.001**
Intergroup	**0.972** (±0.013)	**0.853** (±5.11)	**0.779** (±0.022)	**0.035**	**≤0.001**	**≤0.001**
Significance	**0.568**	**0.475**	**0.422**			
N	**37**	**42**		**34**		

## Discussion

Given the observation of a progressive decrease in intragroup and intergroup reliability in the execution of the Extended TUG test (Table 2) and the significant differences both in execution time and reliability of the observed results, it can be said that the objective of the study was achieved.

### Intragroup and intergroup reliability

Analysis of both intragroup and intergroup reliability in the execution of the Extended TUG test revealed that the results obtained for the groups in the first and second decades were qualitatively excellent [25] and consistent with previous studies conducted on patients within the same age range [26]. However, the ICC values in the G2 decade (65–74 years) were lower (intragroup ICC = 0.858 and intergroup ICC = 0.853) compared with previously published studies, where higher reliability values were observed (ICC = 0.992 and ICC = 0.877, respectively) [22].

No significant differences were found when comparing intragroup and intergroup reliability. This could indicate that the results obtained from the Extended TUG test do not depend on the professionals supervising the test, provided that they have sufficient previous experience for the participant to understand and correctly execute the test.

However, when comparing both intragroup and intergroup reliability between each of the decades, there were significant differences between all the groups (Table 2). The results obtained showed that as the age of the participants increased, the reliability progressively decreased, going from ICC = 0.977 (G1 decade) to ICC = 0.784 (G3 decade) (Table 2). A possible explanation for these differences could be the characteristic pattern of the gait and the mobility of the elderly, which reflect postural and balance changes as psychomotor skills diminish [27]. The prevalence of gait disorders increases progressively as a person ages [28]. Specifically, 85% of people aged 60 years have a normal gait pattern, whereas this figure drops to 20% in those older than 85 years [28]. When referring to age-related changes, some researchers use the term ‘senile gait disorders’ to describe patterns in the elderly that include a slow pace, a broad base and walking cautiously [27], and these changes might justify the lack of precision when performing the Extended TUG test.

### The extended TUG: execution time

The results obtained for the third group (G3: 75–85 years) show an average execution time of 20.53 (± 15.09) seconds (Table 1). These results are in line with the previously observed time of 20.1 (± 11.5) seconds [29] for patients of similar age to those in G3. Similarly, the Extended TUG results observed for G1 (55–64 years) and G2 (65–74 years) – 14.49 (± 5.11) and 17.29 (± 13.87), respectively (Table 1) – are also comparable to the results observed in previous studies, where patients in the G1 group took 12.09 (± 0.51) [22] and 18.9 (± 2.6) seconds [26], respectively.

To the best of our knowledge, no study has been carried out to compare the Extended TUG results of participants between 55 and 85 years of age. When analysing the observed results, significant differences were identified when comparing the three groups used in the present study, with the differences ranging from 2.08 (G1–G2) to 6.04 (G1–G3) (Table 1). The difference observed between the groups could be partly due to the normal physiological changes that occur as the body ages [27]. These changes affect mobility, with mobility defined as the ability to move in the environment easily and without restriction, therefore as the function of other organs that contribute to this complex physiological activity decrease, this reduced function might be reflected in the walking speed [28], which can be evaluated, for example, using the Extended TUG test.

### Clinical implications

The present study is the first to present reference values for the Extended TUG test (as a test of the speed of functional walking) separated by decades (G1 decade: 55–64 years; G2 decade: 65–74 years; G3 decade: 75–85 years) and shows a gradual increase in execution time with advancing age, corroborating the results in the reviewed scientific literature and having implications for geriatric clinical practice. It highlights the need to fragment geriatric functional evaluation according to decades for the elderly, given that the differences in functional capacities are statistically significant, therefore the decades must be separate in their evaluation and treatment in order to adjust the interventions to the characteristics of the patients [30]. The early detection of pre-frail patients by using the Extended TUG test is a very good option for preventive intervention.

### Limitations

Future studies should extend the age of the participants to be able to include participants over the age of 85 years. Moreover, the present study has some weaknesses. For example, it would be interesting to continue to increase sample size in each of the three decades studied and thus be able to offer reference data for each of the decades assessed in this study. Furthermore, it is important to remember that, although the groups were divided into three age groups, no gender separation was made, which would require taking into account the characteristics and differences between men and women when interpreting the results.

## Conclusion

The main conclusion that can be drawn from this study is that the reliability of the execution time of the Extended TUG test progressively decreases as the age of the participant performing the test increases. Similarly, the execution time of the Extended TUG test increases when the average age of the participants is increased. These results, divided by decades, should be taken into account when planning preventive interventions aimed at maintaining or improving the independence of participants within the age range studied.

## Data Availability

The datasets used and/or analysed during the current study are available from the corresponding author on reasonable request.

## Abbreviations

ICC: Interclass correlation
TUG: Timed-Up and Go
